# Fecal microbiota composition affects in vitro fermentation of rye, oat, and wheat bread

**DOI:** 10.1038/s41598-022-26847-y

**Published:** 2023-01-03

**Authors:** Laura Pirkola, Johan Dicksved, Jussi Loponen, Ingela Marklinder, Roger Andersson

**Affiliations:** 1 Department of Molecular Sciences, Swedish University of Agricultural Sciences, P.O. Box 7015, 75007 Uppsala, Sweden; 2 Fazer Sweden AB, P.O. Box 30180, 11343 Stockholm, Sweden; 3 Department of Animal Nutrition and Management, Swedish University of Agricultural Sciences, P.O. Box 7024, 75007 Uppsala, Sweden; 4 Oy Karl Fazer AB, P.O. Box 4, 01230 Vantaa, Finland; 5 Department of Food Studies, Nutrition and Dietetics, Uppsala University, P.O. Box 560, 75122 Uppsala, Sweden

**Keywords:** Microbiome, Dietary carbohydrates, Polysaccharides

## Abstract

Fermentation of dietary fiber by gut microbes produces short-chain fatty acids (SCFA), but fermentation outcomes are affected by dietary fiber source and microbiota composition. The aim of this study was to investigate the effect of two different fecal microbial compositions on in vitro fermentation of a standardized amount of oat, rye, and wheat breads. Two human fecal donors with different microbial community composition were recruited. Bread samples were digested enzymatically. An in vitro fermentation model was used to study SCFA production, dietary fiber degradation, pH, and changes in microbiota. Feces from donor I had high relative abundance of *Bacteroides* and *Escherichia/Shigella*, whereas feces from donor II were high in *Prevotella* and *Subdoligranulum.* Shifts in microbiota composition were observed during fermentation. SCFA levels were low in the samples with fecal microbiota from donor I after 8 h of fermentation, but after 24 h acetate and propionate levels were similar in the samples from the different donors. Butyrate levels were higher in the fermentation samples from donor II, especially with rye substrate, where high abundance of *Subdoligranulum* was observed. Dietary fiber degradation was also higher in the fermentation samples from donor II. In conclusion, fermentation capacity and substrate utilization differed between the two different microbiota compositions.

## Introduction

Consumption of whole grain cereals, rich in dietary fiber, is associated with beneficial health effects, such as lower risk of type 2 diabetes, cardiovascular disease, and colorectal cancer^1^. Arabinoxylan, β-glucan, and cellulose are the major non-starch polysaccharides (NSP) in whole grain cereals^2^. In the Nordics, wheat, rye, and oats are the most commonly consumed whole grain cereals^3,4^. Rye and oats differ in their fiber composition, as oats have a high content of water-soluble β-glucan, whereas rye is rich in arabinoxylan and fructan^2,5^. Whole grain rye and wheat have similar dietary fiber composition, but the content and water solubility of arabinoxylan is higher in rye^5^. Soluble dietary fiber is generally considered to be readily fermentable, whereas water-insoluble fiber, such as cellulose, has lower fermentability^2^. Processing method also seems to affect the fermentability of whole grain NSP^6^.

The gut microbiota is estimated to consist of 10^14^ of microbes that inhabit the gastrointestinal tract, mainly the large intestine^7^. Inter-individual variation in gut microbiota composition is considered greater than changes in microbiota community within an individual. Diet is a major factor affecting gut microbiota composition and functioning, both directly and indirectly^8^. Dietary fiber is the main nutrient source for gut microbes, and fermentation of fiber produces short-chain fatty acids (SCFA), most importantly acetate, propionate, and butyrate^9^. In human physiology, SCFA act as metabolic substrate and as signaling molecules influencing energy homeostasis and the immune system^10–12^.

In 2011, Arumugam et al*.*^13^ published their findings on three different types of microbial communities in the human gut and referred to these as enterotypes, dominated by different genera (*Prevotella*, *Bacteroides*, or *Ruminococcus*) considered to be drivers of community composition. Of these, only the first two have been confirmed in later studies^14^. Enterotypes are suggested to be complex and cannot be explained by human properties, such as age or body mass index. However, the validity of enterotypes has been questioned because they may oversimplify the complexity of human gut microbiota^15^. Metabolic diversity has been observed between the enterotypes, with lower lipolytic and proteolytic fermentation potential in the *Prevotella* enterotype and with the *Bacteroides* enterotype characterized by higher saccharolytic and proteolytic capacity^16^. Recent in vitro studies have shown differences in fermentation between the enterotypes with respect to time, SCFA production, changes in microbiota composition, and preference for different polysaccharides^17–19^. In a study involving in vitro fermentation of fructooligosaccharides (FOS), sorghum arabinoxylan, and corn arabinoxylan, a *Prevotella-*dominated microbiota was found to produce higher levels of SCFA, and propionate production was 2- to threefold higher than for *Bacteroides*-dominated microbiota^17^.

Some previous in vitro studies have shown higher fermentation rate and SCFA production for oat bran compared with rye or wheat bran^20,21^, although in one in vitro fermentation study oat and rye bran were comparable in terms of SCFA production and pH^22^. In human intervention studies, fiber from wheat, rye, or oats has been shown to affect gut microbiota composition and increase the level of fermentation metabolites, but the number of studies is relatively low and the studies have methodological limitations and differences^23^. Nevertheless, current evidence supports the role of intact cereal fiber in promoting microbiota diversity and abundance.

The aim of this study was to investigate the effect of different fecal microbial community compositions from two human donors on in vitro fermentation of oat, rye, and wheat breads in terms of fiber utilization and fermentation outcomes. Bread is a complete food product containing a combination of different fibers, whereas most other in vitro fermentation studies have studied isolated polysaccharides. This study was designed to model gut fermentation of dietary fiber corresponding to a standardized amount of bread with two different microbiotas. The amount of fermentation substrate reflected the dietary fiber content of the breads, and thus differed between rye, oats, and wheat.

## Results

In vitro fecal fermentation experiments were conducted to study SCFA levels, dietary fiber degradation, pH, and changes in microbiota. Fecal samples from two donors with different microbiota composition were used. Two separate experiment occasion per donor resulted into four replicates of each substrate and donor combination. Before experiments, bread samples were enzymatically digested. Study outline is presented in Supplementary Fig. S1 online.

### Chemical composition of bread and fermentation substrates

The three breads differed in chemical composition and especially in the amount and type of dietary fiber (Table 1). Fermentation substrate preparation from bread increased the proportional amount of fiber in all samples, and over 80% of the fiber in bread was recovered (94.2% for oats, 87.8% for rye, 82.7% for wheat). Starch was almost completely removed from the samples (< 0.5% recovered) and the amount of protein and lipids was lowered, with approximately 40% of proteins and 80% of lipids removed during the process. The ratio of insoluble and soluble fiber was only slightly affected by the substrate preparation process (Table 1). The proportion of fiber in the substrates varied from 23.8% in wheat to 46.5% in rye, and the calculated amount of fiber in the fermentation experiments was 0.55 g in oats, 1.09 g in rye, and 0.25 g in wheat, respectively.

**Table 1:** Nutritional composition (% of dry matter) and the ratio of insoluble-to soluble dietary fiber of breads and fermentation substrates derived from the breads, analyzed in duplicate samples.

	Bread			Substrate		
	Oats	Rye	Wheat	Oats	Rye	Wheat
Protein	14.8	10.5	11.9	28.5	19.6	35.2
Lipids	14.0	1.9	3.4	7.9	1.1	5.4
Starch	35.8	47.7	57.9	0.3	0.3	0.1
Sugars total	4.9	1.7	2.9	13.3	8.0	12.4
Glucose	0.9	0.3	0.1	5.8	4.8	3.0
Fructose	1.6	0.9	0.3	3.7	2.3	1.3
Sucrose	0.3	0.0	0.2	0.6	0.1	0.7
Maltose	2.1	0.5	2.3	3.3	0.7	7.4
Dietary fiber^a^	11.3	17.3	5.2	33.3	46.5	23.8
Insoluble	7.0	12.1	3.2	18.3	31.8	13.8
Soluble	4.3	5.2	2.0	15.0	14.7	10.0
Fructan	0.1	1.5	0.4	0.2	2.4	1.2
Arabinoxylan^b^	3.4	8.3	1.6	11.2	24.8	8.6
β-Glucan	3.2	1.9	1.0	9.8	5.8	6.0
Raffinose	0.1	0.0	0.0	0.1	0.0	0.0
*Insoluble-to-soluble fiber ratio*	1.6	2.3	1.6	1.2	2.2	1.4

^a^Calculated as the sum of fructan (AOAC Method 999.03) and dietary fiber analyzed by AOAC Method 994.13.

^b^Calculated from arabinose, xylose and galactose residue values (analyzed by AOAC Method 994.13) assuming that arabinose to xylose ratio is 0.69 in arabinogalactan.

### Microbiota composition

The microbiota composition of fecal samples used in the fermentation experiments differed between the two donors (Supplementary Fig. S2 online). Analysis of the fecal samples from donor I showed high relative abundance of the genera *Bacteroides*, *Christensenellaceae* R-7 group, *Blautia*, and *Akkermansia*. In contrast, the fecal samples from donor II had high relative abundance of the genera *Prevotella*, *Subdoligranulum*, and *Bacteroides*.

**Figure 1:**
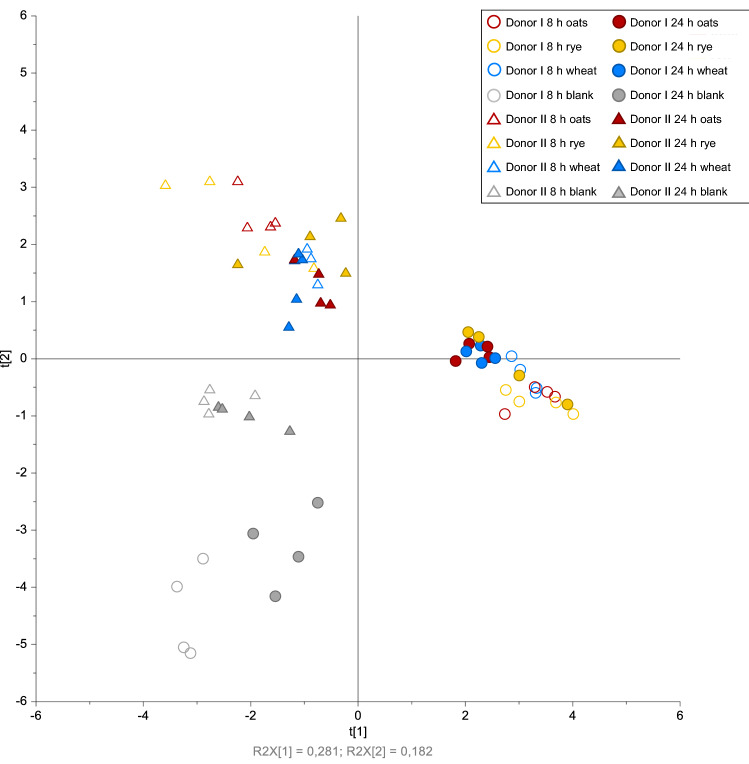
Principal component analysis (PCA) scores plot of the 20 most abundant genera in fermentation samples. In PCA, the first principal component (PC1, horizontal) accounts for the largest variance in the dataset. Residuals R2X (1) and R2X (2) indicate the amount of variation in the model described by PC1 and the second principal component PC2 (vertical), and t(1) and t(2) are co-ordinates of the PCA projection. Blank indicates samples without substrate, and oats, rye and wheat indicate samples with fermentation substrate.

**Figure 2:**
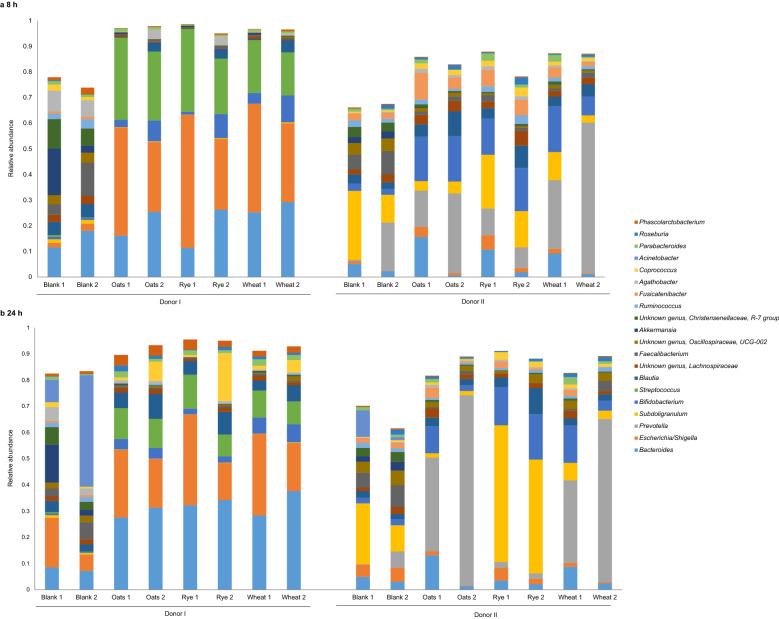
The 20 most abundant microbial genera in (**a**) 8 h fermentation samples, and (**b**) 24 h fermentation samples. Each bar represents mean of replicates (n = 2) from one fermentation experiment occasion (1 and 2). Blank indicates samples without substrate, and oats, rye and wheat indicate samples with fermentation substrate.

In principal component analysis (PCA), the microbiota composition of the fermentation samples at 8 h and 24 h was clearly separated for the two donors based on the first principal component (Fig. 1). Moreover, blank samples were separated from the samples with substrate, but the fermentation substrates were not clearly separated from each other. Shifts in relative abundance between genera were observed during the 24 h fermentation (Fig. 2). In the fermentation samples with fecal microbiota from donor I (hereafter referred to as donor I samples), all three fermentation substrates gave similar microbiota composition, with high relative abundance of *Bacteroides*, *Escherichia/Shigella*, and *Streptococcus* at 8 h and 24 h. The relative abundance of *Bacteroides* increased from 8 to 24 h, while the relative abundance of the other two genera decreased. In the fermentation samples with fecal microbiota from donor II (hereafter referred to as donor II samples), high relative abundance of *Subdoligranulum* (0.48 ± 0.15) was found for the rye substrate after 24 h fermentation, compared with oats (0.016 ± 0.0052) and wheat (0.049 ± 0.024). In the donor II samples, the highest relative abundance of *Bifidobacterium* was detected for the rye substrate, while the samples with oat and wheat substrates had high relative abundance of *Prevotella* (0.54 ± 0.21 for oats and 0.46 ± 0.18 for wheat) compared with rye substrate (0.022 ± 0.016) at 24 h. Similar, but less pronounced, differences between the substrates were observed at 8 h.

The analysis of similarities (ANOSIM) showed a clear difference in microbiota composition between the donors (R = 0.918, p < 0.001). In the donor I samples, experiment occasion had the highest effect on the dissimilarities in microbiota composition when samples at the 8 h and 24 h time points were analyzed separately (R = 0.787, p = 0.003 for 8 h; and R = 0.820, p = 0.002 for 24 h, respectively), followed by the effect of time point (Supplementary Table S1 online). Substrate effect on dissimilarities was not significant in donor I samples. In the donor II samples, substrate had the highest effect on dissimilarities in microbiota composition, especially at 24 h (R = 0.676, p = 0.003). The time point effect was small, and the experiment occasion effect was significant at 8 h but not at 24 h.

### SCFA and branched-chain fatty acid (BCFA) levels

**Figure 3:**
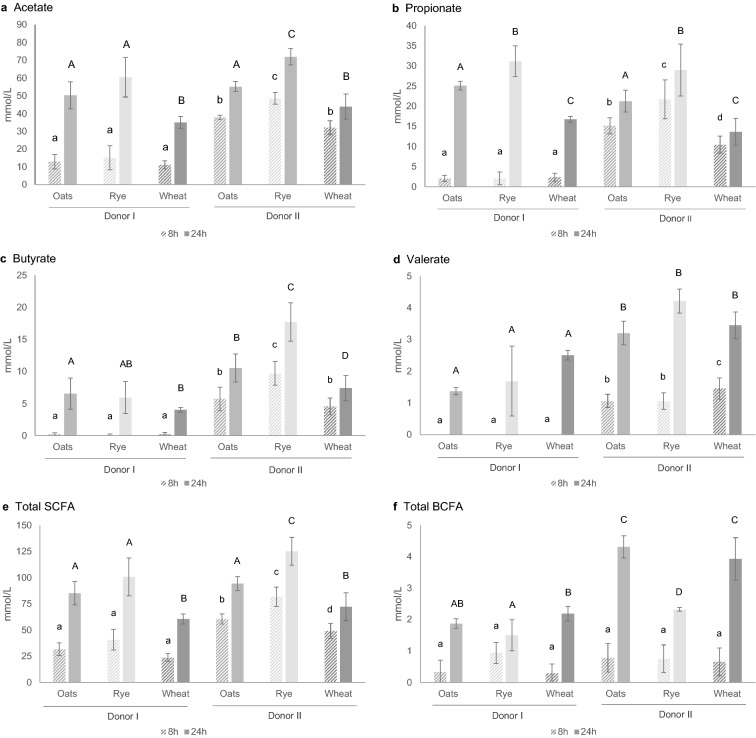
(**a**–**e**) Short-chain fatty acid and (**f**) branched-chain fatty acid concentrations after 8 h and 24 h of fermentation (mean of n = 4 replicates, error bars indicate s.d.). Different letters above bars indicate statistically significant differences in pairwise comparisons for each substrate-donor combination (lowercase 8 h, and uppercase 24 h, p < 0.05). Oats, rye and wheat indicate samples with fermentation substrates (*BCFA* branched-chain fatty acids, *SCFA* short-chain fatty acids).

After 8 h of fermentation, the levels of acetate, propionate, butyrate, and valerate were higher in the donor II samples (p < 0.0001) (Fig. 3). A small interaction between donor and substrate type in the statistical model was observed for acetate, propionate, and butyrate levels. Significant differences between the substrates were observed in most pairwise comparisons of the donor II samples, with the highest SCFA levels in rye and lowest in wheat (except for valerate). No differences between the substrates were observed for the donor I samples at 8 h. The levels of total SCFA were aligned with the individual SCFA results at 8 h.

After 24 h fermentation, similar acetate and propionate levels were observed for the samples from both donors. An interaction between donor and substrate type in the statistical model was not detected for acetate or propionate level at 24 h, whereas a small interaction was detected for butyrate level, which was explained by high butyrate levels in rye substrate in donor II samples. Butyrate levels were higher in the donor II samples after 24 h of fermentation (p < 0.0001). Moreover, in pairwise comparison the 24-h butyrate levels in the donor II samples differed between all fermentation substrates, with rye in particular contributing to high butyrate levels. In the donor I samples, butyrate levels were similar between oats and rye. For valerate, a difference in 24-h levels was seen in comparison between the donors, but not between the substrates.

There was no difference in BCFA levels at 8 h between the samples from the different donors, or between the substrate types. The BCFA levels were higher in the donor II samples after 24 h of fermentation (p < 0.0001). A small interaction between donor and substrate type in the statistical model was observed for BCFA levels at 24 h. In the donor II samples, the oats and wheat substrates gave higher BCFA levels than rye. Lactate was detected in the donor I samples at 8 h (16.97 ± 1.40 mmol/L for oats, 23.07 ± 1.27 mmol/L for rye, and 9.59 ± 0.75 mmol/L for wheat) but not at 24 h.

In the ANOSIM, SCFA levels were dissimilar between the donors (R = 0.210, p = 0.003), and the effect of substrate on the dissimilarities in SCFA was higher in the donor II samples (Supplementary Table S1 online). Experiment occasion effect on SCFA dissimilarity was significant only in the donor I samples at 8 h. Time point (8 h vs 24 h) effect was seen in the both donor samples, and was high in the donor I samples (R = 0.999, p < 0001).

### Recovery of NSP sugar residues

**Figure 4:**
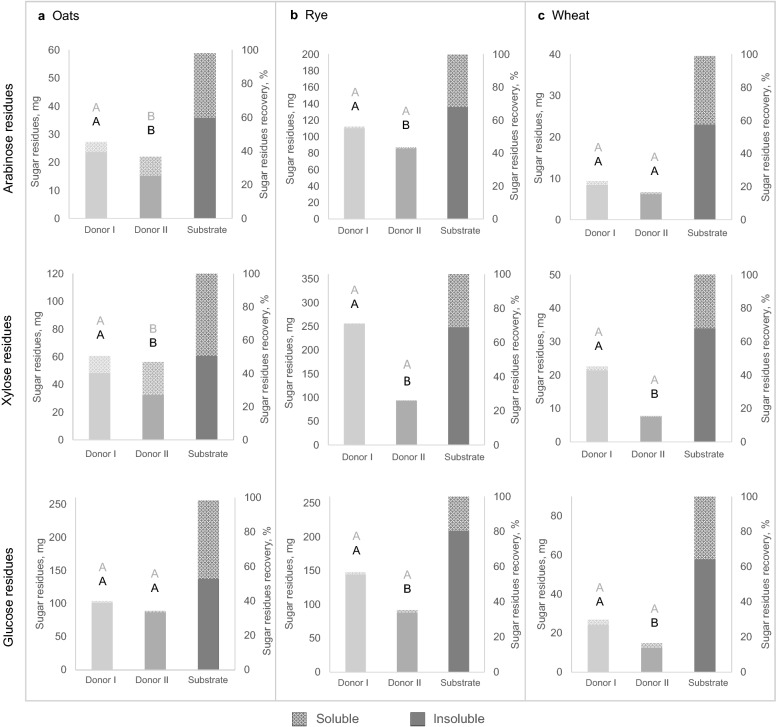
Amount and percentage recovery of arabinose, xylose, and glucose residues after 24 h of fermentation, and sugar residue composition in substrates in (**a**) oats, (**b**) rye and (**c**) wheat. Sugar residue amount varied between the substrates due to differences in fiber composition and substrate amount. Each bar includes soluble and insoluble sugar residues separately (mean of n = 4 replicates), and different letters (grey for soluble and black for insoluble sugar residues) above bars indicate statistically significant differences (p < 0.05) in pairwise comparisons between the two donor samples. Oats, rye and wheat indicate samples with fermentation substrate.

The lowest recovery of insoluble sugar residues was observed for wheat samples, for which the amount of sugar residues in the fermentation substrate was also lower than in rye and oat substrates (Fig. 4). Recovery of insoluble arabinose residues was lower for the donor II samples compared with donor I samples with oat and rye substrate. In addition, insoluble xylose residue recovery was lower for the donor II samples, and the difference between the two donor samples was substantial, especially for the rye substrate (25.5 vs 70.2%, p < 0.0001). A small interaction in the statistical model between substrate and donor was observed for insoluble xylose residues. Moreover, lower recovery of insoluble glucose residues was observed for the donor II samples (p < 0.0001), but the difference in oat substrate was not significant in pairwise comparison. Prominent amounts of soluble arabinose and xylose residues were detected only in the oat substrate samples after fermentation, and sugar residue recovery was lower in the donor I samples. A moderate interaction between donor and substrate type in the statistical model was observed for soluble arabinose and xylose and was explained by higher recovery of these sugar residues in the donor II oat substrate samples. There was no difference in recovery of soluble glucose residues between the fermentation samples with fecal microbiota from the two donors or any substrate. Only a small amount of insoluble sugar residues was observed in the blank samples after fermentation (2.1 ± 1.6 mg).

Lower recovery of insoluble (p = 0.0001) and soluble (p = 0.0002) mannose residues was observed in donor II samples compared with donor I samples (Supplementary Table S2 online). The amount of insoluble galactose residues was approximately the same or higher after 24 h of fermentation when compared with the sugar residue levels in the substrate and did not differ between the fermentation samples with fecal microbiota from the two donors. Recovery of soluble galactose residues was lower in the donor II samples for oat (p < 0.0001) and wheat (p < 0.001) substrate, but higher for rye substrate (p = 0.01).

### pH

The inoculate pH was 7.06 ± 0.02. Samples containing substrate had lower pH values than blanks at 8 h and 24 h (Table 2). A moderate interaction between donor and substrate type was detected in the statistical model. The pH was lower in the donor II samples than the donor I samples for rye substrate at 8 h (p < 0.0001) and at 24 h (p < 0.001) but did not differ for the other substrates. In the donor II samples, pH differed between all substrates in pairwise comparisons at both 8 and 24 h and was lowest for the rye substrate (p < 0.05).

**Table 2:** Changes in pH during the fermentation experiments (mean ± s.d.). Blank indicates samples without substrate, and oats, rye and wheat indicate samples with fermentation substrates.

	Donor I		Donor II	
	8 h	24 h	8 h	24 h
Blank	7.22 ± 0.02	7.00 ± 0.18	7.20 ± 0.01	7.12 ± 0.03
Oats	6.96 ± 0.05	6.89 ± 0.14	6.94 ± 0.06	6.84 ± 0.05
Rye	6.98 ± 0.13	6.75 ± 0.17	6.69 ± 0.07	6.45 ± 0.17
Wheat	7.05 ± 0.03	6.99 ± 0.16	7.06 ± 0.04	7.06 ± 0.04

## Discussion

This study was designed to model gut fermentation of dietary fiber corresponding to a standardized amount of bread, and thus the amount of fermentation substrate used depended on the fiber content of the bread. SCFA levels, and fiber degradation were higher for the samples inoculated with microbiota dominated by *Prevotella*, *Subdoligranulum* and *Bacteroides* (donor II) than for the samples inoculated with microbiota high in *Bacteroides, Christensenellaceae* R-7 group, *Blautia*, and *Akkermansia* (donor I). SCFA levels were highest for the rye substrate, probably due the higher fiber content.

The microbiota composition in the fermentation samples with fecal microbiota from donor I was similar between all three substrates, with the highest relative abundance of *Bacteroides* after 24 h of fermentation. Interestingly, the relative abundance of *Escherichia/Shigella* increased notably during fermentation in donor I samples, as the relative abundance of the genus was very low in the fecal samples. *Escherichia coli* has a very robust growth mechanism^24^, and our hypothesis is that the genus had a competitive benefit in the beginning of the fermentation. Microbiota composition in the donor II samples differed between the substrates, which was also observed in the ANOSIM. The abundance of *Subdoligranulum* was high for the rye substrate, whereas the relative abundance of *Prevotella* was high for the oat and wheat substrates, especially after 24 h of fermentation. The highest relative abundance of *Bifidobacterium* was detected for the rye substrate, which is in line with findings by Eriksen et al.^25^ that an 8-week rye diet can increase the relative abundance of *Bifidobacterium*. In vitro studies have demonstrated that rye bran and soluble oat fiber can enhance the growth of *Bifidobacterium*, and that rye bran can enrich *Prevotellaceae*^26^. A study by Chen et al.^17^ comparing fermentation of different fiber types in the *Bacteroides* and *Prevotella* enterotypes showed a higher diversity of taxa that responded to fiber substrates in the *Bacteroides* enterotype, whereas in the *Prevotella* enterotype, *Prevotella* was the only taxa to increase on the fiber substrates. This was partly confirmed by results in the present study, since either *Prevotella* or *Subdoligranulum* had distinctly high relative abundance after 24 h in the donor II samples depending on the substrate, whereas high abundance of any single genus was not observed in the donor I samples. The connection between rye and *Subdoligranulum* observed in the present study needs to be verified in future studies.

At 8 h, SCFA levels were low in the fermentation samples with fecal material from donor I, but after 24 h of fermentation, only butyrate levels differed between the samples with fecal microbiota from the two donors. The higher butyrate production from rye substrate in the donor II samples is likely explained by differences in microbiota composition. For BCFA, no differences were observed at 8 h, but after 24 h, the levels were higher in the donor II samples. The levels of BCFA, which are metabolites of branched-chain amino acid fermentation in gut and biomarkers of protein fermentation, were higher for the oat and wheat substrates, reflecting the higher protein content of these substrates. It is also possible that higher fiber fermentation inhibited protein fermentation in the samples with rye substrate^27^. Protein fermentation causes changes in microbiota composition and metabolite production that can have negative health effects, but the evidence is still controversial, and especially the physiological role of BCFA is not well known^28^. The strong effect of time point on SCFA in the donor I samples observed in the ANOSIM can be explained by lactate production that was observed only at 8 h time point.

Rye gave the highest SCFA levels in this study, but previous in vitro studies have shown high fermentability and SCFA levels for oats. In one study, carbohydrates from oat bran fermented at a higher rate and SCFA production was slightly higher than with carbohydrates from rye and wheat bran^20^. In another in vitro fermentation study^21^, oat bran increased propionate and acetate production. On the other hand, in an in vitro fermentation study conducted by Roye et al.^22^, oat and rye bran were comparable in terms of SCFA production. In that study, bran was removed from residual endosperm without removal of fructan and water-extractable arabinoxylan, which, according to the authors, explained the better fermentability of rye than seen in other studies. Fructan was recovered also in the present study, which probably improved the fermentability of the rye substrate.

Acetate is produced by many bacterial groups in the human colon, but bacteria that produce propionate and butyrate are fewer and there are several pathways of SCFA metabolism that vary between bacterial groups^29^. High relative abundance of *Subdoligranulum* can explain the high butyrate production seen for the donor II samples compared with the donor I samples in the present study, as it has been shown that certain *Subdoligranulum* species form butyrate through the butyrate kinase route^30^. Chen et al.^17^ found that the *Prevotella* enterotype produced higher levels of SCFA with FOS and arabinoxylan, and that propionate production was 2–3 times higher than for the *Bacteroides* enterotype. Yang et al.^31^ found that *Bacteroides* was positively correlated with propionate production in in vitro fermentation. Yu et al.^19^ reported higher butyrate and propionate levels in *Prevotella* than *Bacteroides* enterotype in in vitro fermentation of pea cell wall polysaccharides. In the present study, donor II samples had high butyrate levels, and propionate levels were similar between the two donor samples.

In the present study, the fiber composition in post-fermentation samples is reported as recovery of insoluble and soluble sugar residues. These sugar residues originate from grain NSPs, most importantly arabinose and xylose from arabinoxylan, soluble glucose from β-glucan, insoluble glucose from cellulose, mannose from glucomannan, and galactose from arabinogalactan^2^. Low recovery of sugar residues indicates high utilization of substrate fiber by bacteria during fermentation. Recovery was calculated from total sugar residues in the substrate (i.e. the sum of insoluble and soluble sugar residues), based on the hypothesis that some of the insoluble sugar residues could have been solubilized during fermentation. Some differences in sugar residue recovery were detected between the samples with fecal microbiota from the different donors. Lower recovery of insoluble arabinose and xylose was observed in the donor II samples with oat and rye substrate which indicates more effective utilization of insoluble arabinoxylan, possibly due to higher fermentation capacity of the bacteria in donor II samples. Interestingly, only oat substrate samples contained prominent amounts of soluble arabinose and xylose sugar residues. It is possible that part of the insoluble arabinoxylan was solubilized during fermentation of oat substrate, but not rye or wheat substrate. Lower recovery of insoluble glucose residues from the rye and wheat substrates was observed in the donor II samples, indicating more effective fermentation of insoluble β-glucan and possibly cellulose. For samples with oat substrate, differences were not detected in soluble or insoluble glucose residues, which is probably explained by the high content of easily fermentable soluble β-glucan in oats. In general, the amount of soluble sugar residues was very low in most samples after fermentation, which indicates that these were more readily fermentable than the insoluble sugar residues.

The chosen method of processing bread samples to fermentation substrate had certain strengths and limitations. It was successful to remove starch and restore fiber, but only around half the protein was removed for all sample types. The process did not substantially decrease the sugar content of samples. In similar fermentation studies, in vitro digestion with mammalian enzymes is commonly used. The method used in the present study was chosen because it decreased the starch content to almost zero and enabled retention of fructan, which is usually removed during in vitro digestion of food or grain material before fermentation^22^. There were some weaknesses with the method. First, retention of fructan retained also other ethanol-soluble molecules, such as glucose, in the substrate. Second, the method is not alike to the digestion process in the human small intestine and the enzymes used were not of mammalian origin.

A limitation as regards dietary fiber recovery is that the polysaccharide levels in the fecal inoculates were not measured. However, the fiber amount in blank samples after fermentation was minimal and no gas production was detected, which indicates that the amount of fiber originating from the inoculate was negligible. The samples used for fiber analysis after fermentation experiments were autoclaved before analysis to avoid any pathogen risk, which may have affected the fiber structures present. However, the soluble and insoluble fractions were separated before autoclaving, and thus the treatment did not affect the ratio of insoluble to soluble fiber in the fermentation samples, and effects on sugar residue content are unlikely.

The aim in this study was to mimic colonic fermentation of three bread products with different fiber content, and thus the amount of fermentation substrate and the fiber amount differed between the three substrates. This difference in fiber amount limited between-substrate comparisons, since the amount of available fermentable compounds affects production of SCFA and BCFA. The difference in fiber amount also limited comparison of the results with those of studies in which the fiber or substrate amount was similar for different grains.

The low SCFA levels at 8 h, and low gas production observed during the first 8 h of the fermentation experiments in the donor I samples indicate that the fermentation process started more slowly than in the donor II samples. This might relate to differences in fermentation capacity of different bacterial taxa but can also have been caused by the number of actively growing bacteria in the sample. Viable cell counts were not conducted on fecal samples or inoculates, which is a limitation. Moreover, the handling of fecal samples and oxygen exposure could have affected the results, since donors collected a sample shortly before each experiment, but the time between sample collection and fermentation was not standardized. The fecal inoculate was not processed under strictly anaerobic conditions, although oxygen exposure was minimized. Processing of the sample to produce inoculate slightly changed the relative abundance of certain genera.

Since previous studies have reported difference in fermentation capacity between the enterotypes, we used *Bacteroides* and *Prevotella* genera as a premise to find fecal donors with contrasting microbiota composition. We did not find donors with clear *Bacteroides* or *Prevotella* dominated microbiotas, but the two donors had different microbiota composition and fermentation outcomes differed between the donor samples. There was some variation in fecal sample microbiota composition between the two experiment occasions, especially in donor II. However, the microbiota composition was similar between the experiment occasions after 24 h fermentation in the samples with substrate, and the replicates showed overall good repeatability within and between experiments. In the ANOSIM, a strong effect of the experiment occasion on dissimilarities in microbiota was observed in the donor I samples, but in the donor II samples it was significant only at 8 h but not at 24 h. In donor II samples, substrate had a pronounced effect on microbiota composition.

In most of previous in vitro fermentation studies, the fermented material was grain bran or isolated fiber, not a complete food product containing a combination of different fiber structures. A strength of this study is that the breads used were existing commercial consumer products or similar. The specially produced refined wheat bread containing oat endosperm flour was used in the present study because it was developed as a placebo bread for a clinical trial within the same project. Oat endosperm flour has high starch content and contains 4.0–5.0% of dietary fiber^32^, and the amount of oat endosperm flour in the bread was only 25% of flour ingredients. As the aim of the study was not to compare rye and oat bread to whole grain wheat bread, the wheat bread was made of refined flours and had low fiber content.

Gut microbiota composition differs between individuals, which can affect gut fermentation, as shown in this and previous studies. SCFA outcomes, which depend on microbiota-fiber interactions, can lead to differences in health effects between individuals. SCFA play an important role in human physiology and energy balance, and studies with animal models have even identified a role of SCFA as mediators in the gut-brain axis, the bi-directional communication pathway between the gut and the brain^33^. Conclusive evidence from human trials that different fiber structures promote SCFA production in individuals with different gut microbiota composition can lead to more personalized dietary recommendations for prevention and treatment of different diseases and conditions.

In conclusion, in 24 h in vitro batch culture fermentation experiments, there were clear differences in SCFA production and in fiber degradation between samples with fecal microbiota from two donors with different fecal microbiota composition. Differences in butyrate, propionate, and acetate concentrations were found between oat, rye, and wheat bread substrates, especially in donor II fermentation samples. Microbiota composition changed during the fermentation experiments. The relative abundance of *Bacteroides* and *Escherichia/Shigella* increased in the donor I samples, while the relative abundance of *Prevotella*, *Subdoligranulum* and *Bifidobacterium* increased in the donor II samples. These results indicate contrasting fermentation capacity and substrate utilization potential between different microbiota profiles in the human gut. This suggests that differences in microbiota profile could in part explain intra-individual differences in diet-related health outcomes, due to differences in metabolite production.

## Methods

### Bread samples

Three different bread products were used: a commercial whole grain rye bread, a commercial whole grain oat bread, and a refined wheat bread containing oat endosperm flour (25% of flour). Ingredient lists and other details are presented in Supplementary Table S3 online. The breads were freeze-dried for 5 h at 30 °C and 0.01 mbar, followed by approximately 20 h at 0 °C and 1.5 mbar. Dried samples were milled in a laboratory mill to pass a 0.5 mm screen. After milling, the bread samples were stored at − 20 °C.

### Removal of lipids, available starch, and savinase-degradable protein from bread

Freeze-dried and milled bread samples (35 g) were weighed into centrifuge bottles. To remove lipids, each sample was mixed with 50 mL of n-heptane, vortexed twice for 2 min, and centrifuged (10 min, 2000×*g*), after which the heptane layer was discarded. For rye and wheat bread samples, this heptane washing was repeated once, while for the oat bread samples it was repeated twice, after which the residues were air-dried.

To extract fructan, the dried residue was mixed with 250 mL of ethanol (80% v/v) and incubated at 80 °C for 45 min with magnetic stirring (500 rpm). After incubation, the sample was centrifuged (10 min, 1000×*g*) and the supernatant was collected. Thereafter, the sample was washed three times by adding 30 mL of ethanol (80% v/v), mixing, and centrifuging (10 min, 1000×*g*), with the supernatant collected after each centrifugation. Ethanol was removed from the pooled supernatants by vacuum rotor evaporation, and the unevaporated residue containing fructan was mixed with 50–100 mL of deionized water and frozen at − 20 °C.

To remove starch, an amylolytic treatment was carried out. The solid residue from ethanol washing was dried overnight at 40 °C and dispersed in 175 mL of acetate buffer (0.1 M, pH 5.0 and 5 mM CaCl_2_) in a bottle. Then 1.75 mL thermostable α-amylase (3000 U/mL) was added and the sample was incubated at 100 °C for 60 min, with mixing three times during incubation. The solution was cooled to 40 °C, followed by addition of 10.5 mL of amyloglucosidase solution (140 U/mL), and overnight incubation at 60 °C in a shaking water bath. For oat bread samples, 25 mL of acetate buffer were added before amyloglucosidase treatment, to ensure homogeneous mixing.

To remove proteins, the dispersion was cooled to room temperature, and 1.85 mL Savinase (≥ 16 U/g, Sigma-Aldrich) was added, followed by incubation for 3 h at 50 °C in a shaking water bath. Thereafter, the sample was cooled to room temperature and ethanol (99.5% v/v) was added to make 80% (v/v) ethanol solution. The solution was shaken vigorously for 2 min, centrifuged (15 min, 1000×*g*), and the supernatant liquid was discarded. The pellet was washed three more times with 60 mL of ethanol (80% v/v). The solid residue was dried overnight at 40 °C, and mixed with the extract containing fructan. The mixture was frozen, freeze-dried, and milled as described above, and stored at − 20 °C.

### Chemical analysis of bread and substrate samples

Chemical composition of bread samples and of substrates derived from the bread samples was analyzed in duplicate, with the results presented on a dry weight basis after drying at 105 °C for 16 h. Dietary fiber content and composition were analyzed according to the AOAC Method 994.13^34^, with previously described modifications^35^ to analyze the extractable and non-extractable dietary fiber separately. For the analysis of substrates, sample amount of 75 mg was used. The β-glucan content was analyzed with K-BGLU kit (Megazyme) as described previously^36^. The fructan content was determined with a K-FRUC kit (Megazyme) as previously described^37^, with modifications described in Supplementary methods online. Starch content was analyzed enzymatically according to a previously published method^38^. Protein content was analyzed according to the Kjeldahl method^39^ as Kjeldahl-N × 6.25. Fat content was analyzed as described previously^40^. The concentration of glucose, fructose, sucrose, maltose, and raffinose was analyzed as described previously (modified)^41^.

### Study subjects and fecal sample collection

Healthy study subjects (n = 10) were recruited and screened according to exclusion and inclusion criteria (Supplementary Table S4 online) to find two fecal donors with contrasting gut microbiota composition. The Swedish Ethical Review Authority approved the study protocol (application number 2019-04229) and the study was performed following the relevant guidelines and regulations. All study subjects signed an informed consent before being enrolled.

All study subjects collected a screening fecal sample using *EasySampler for stool collection* (GP Medical Devices) and a small sample tube. The screening fecal samples were stored at − 80 °C. For rapid screening of donor microbial profile, the molecular fingerprinting method terminal restriction fragment length polymorphism (T-RFLP) was used, according to a previously described protocol^42^. The T-RFLP data generated by screening samples from all study subjects were evaluated in order to identify two donors with different microbial community composition, with regard to terminal restriction fragments associated with *Bacteroides* and *Prevotella* in previous studies. Based on the T-RFLP data, two donors with contrasting microbiota composition were selected to provide fecal samples for the in vitro experiments.

Fecal samples for the fermentation experiments were collected within two hours before each experiment (including sample processing described below). The donors collected sample at home using an *EasySampler* and a plastic beaker (500 mL) with a sealed cap for collecting minimum 30 g of feces, and the samples were stored at room temperature until the experiment. Approximately 1 g of each fermentation fecal sample was frozen and stored at − 80 °C for microbiota composition analysis.

### In vitro fermentation experiments

Four batch fermentation experiments were conducted with fecal samples from each donor at two separate occasions, resulting in four replicates of each substrate and donor combination The amount of fermentation substrate was energy-standardized between the breads. In addition, inulin (Merck KGaA) was used in control samples to monitor the fermentation process. Substrate (1.65 g of oats, 2.35 g of rye, 1.03 g of wheat substrate, or 1.00 g of inulin) was added to fermentation bottles. Thereafter, 50 mL of buffer (8.5 g NaHCO_3_, 5.8 g K_2_HPO_3_, 0.5 g (NH_4_)_2_HPO_4_, 1.0 g NaCl, 0.5 g MgSO_4_·7 H_2_0, 0.01 g FeSO_4_·7 H_2_0, 0.1 g CaCl_2_ to 1 L of deionized water, pH 7.0)^43^ were added to each bottle and to two bottles without substrate (blank controls). All bottles were treated with CO_2_ gas until addition of inoculate. Inoculate was produced by mixing fecal sample (20 g) with buffer (1500 mL) in a bottle with CO_2_ gas treatment, to obtain 1% (w/v) solution for the fermentation. The fecal slurry was filtered through a kitchen sieve and one layer of polyester filter cloth, and 50 mL were immediately added to the bottles containing buffer and substrate or blank controls. The bottles were closed and incubated at 37 °C for 24 h. Bottle contents were mixed with a motor stirrer throughout the experiment (60 s stirring, 60 s break). Gas production was measured throughout the experiment using the Gas Endeavor system (Bioprocess Control) to follow the fermentation process.

At time points 8 h and 24 h, liquid (5 mL) was collected from each bottle with a syringe and divided into three 1 mL-aliquots, and pH was measured. Aliquots were stored at − 20 °C for later analysis of microbiota composition and volatile compounds. After 24 h of fermentation and sample collection, the fermentation residue material was centrifuged (5 min, 5000×*g*), and the supernatant liquid was separated from the pellet. The supernatant and pellet were autoclaved at 125 °C for 15 min, frozen to − 20 °C, freeze-dried as described above, and stored at − 20 °C.

### Analysis of fermentation samples

Fecal samples, inoculates, and fermentation samples at time points 8 h and 24 h were analyzed for microbiota composition with 16S rRNA gene sequencing as described in Supplementary methods online. Acetate, propionate, butyrate, valerate, BCFA, and lactate concentrations were analyzed as described previously^44^.

Dietary fiber amount and composition after fermentation was analyzed using the fermentation residue material. Pellet composition was analyzed to estimate insoluble fiber degradation, and supernatant composition to estimate soluble fiber degradation. Dietary fiber was analyzed according to the AOAC Method 994.13^34^ with published modifications^35^, and additional modifications described in Supplementary Methods online. All samples were analyzed in duplicate, and results are presented on a dry weight basis, after drying at 105 °C for 16 h.

### Data processing and statistical analysis

To estimate fiber degradation, the amount of each insoluble and soluble sugar residue in fermentation samples was calculated as a percentage of total sugar residues (insoluble plus soluble) in the fermentation substrate. Total SCFA content at 8 h and at 24 h was calculated as the sum of acetate, propionate, butyrate, and valerate, while total BCFA content was calculated as the sum of isobutyrate and isovalerate. The microbiota composition data were analyzed to determine relative abundance on genus level. The cut-off value for data was set at 0.9% of average relative abundance, which represented 85% of total genera abundance. These comprised the 20 most abundant genera in the dataset and were used in further data analysis.

PCA was used for exploratory data analysis of microbiota data (Simca v. 16, Umetrics). For PCA modelling, the data were scaled (univariate scaling) and log-transformed. Analysis of similarities (ANOSIM) was used to statistically test for multivariate differences in microbiota and SCFA data between categorical variables (donor, substrate, time point and experiment occasion) (PAST v. 4.11^45^). The ANOSIM was based on Bray Curtis metrics where the effect of substrate, time and experiment was evaluated for each donor separately. SCFA and BCFA levels, fiber degradation, and pH were statistically compared between the fermentation samples with fecal microbiota from the two donors and between the different fermentation substrates, using a generalized linear fixed-effects model and two-way ANOVA with interaction (RStudio v. 1.2.5019^46^). The generalized linear model included the following fixed-effects variables: donor, substrate, the interaction between donor and substrate, and experiment occasion. Homoscedasticity and normality of residuals in each linear model were checked and, if either was detected, the response variables were log-transformed. This was the case for 8 h butyrate, 8 h valerate, and soluble glucose residues. Statistically significant interactions between donor and substrate variables were examined with an interactions plot, and post hoc pairwise comparison of estimated marginal means was conducted (R package *emmeans*^47^). All analyses were adjusted for multiple comparisons (Tukey’s HSD). Inulin controls and blank samples were not included in the statistical analyses. Descriptive statistical analysis was conducted in Microsoft Excel.

## Supplementary Information

Supplementary Information.

## Data Availability

16S rRNA gene sequences of fermentation samples generated and analyzed during the current study are available in the Sequence Read Archive (SRA) repository, accession number PRJNA853911. The other datasets generated in the study are available from the corresponding author on reasonable request.
