# Supplementary material for: Fecal microbiota composition affects in vitro fermentation of rye, oat, and wheat bread
Source: Sci Rep. 2023 Jan 3;13:99. doi: 10.1038/s41598-022-26847-y (PMC9810601; doi:10.1038/s41598-022-26847-y)
Supplement: Supplementary file 1 — Supplementary Information. [file 41598_2022_26847_MOESM1_ESM.pdf]

Supplementary Information

Fecal microbiota composition affects *in vitro* fermentation of rye, oat, and wheat bread

Laura Pirkola, Johan Dicksved, Jussi Loponen, Ingela Marklinder, Roger Andersson

[1]Department of Molecular Sciences, Swedish University of Agricultural Sciences, P.O. Box 7015, Uppsala, 75007, Sweden

[2]Fazer Sweden AB, Stockholm, Sweden

[3]Department of Animal Nutrition and Management, Swedish University of Agricultural Sciences, P.O. Box 7024 Uppsala, 75007, Sweden

[4]Oy Karl Fazer AB, P.O. Box 4, Vantaa, 01230, Finland

[5]Department of Food Studies, Nutrition and Dietetics, Uppsala University, P.O. Box 560, Uppsala, 75122, Sweden

[*]laura.pirkola@slu.se

Supplementary table S1. Model variables, R-values and p-values for Analysis of similarities (ANOSIM) of microbiota and SCFA. The effect of substrate, sample time point (8 h vs 24 h) and experiment occasion was evaluated for each donor separately. Statistically significant R-values are bolded; an R-value close to 1.0 suggests dissimilarity between groups. (SCFA, short chain fatty acids).

Target data	Donor	Categorical variable	Sample time	R-value	p-value
Microbiota	Donor I	Substrate	8 h	0.014	0.382
			24 h	0.132	0.167
			8 h & 24 h	-0.018	0.549
		Experiment occasion	8 h	**0.787**	**0.003**
			24 h	**0.820**	**0.002**
			8 h & 24 h	**0.239**	**0.005**
		Time point	8 h & 24 h	**0.606**	**<0.001**
	Donor II	Substrate	8 h	**0.336**	**0.036**
			24 h	**0.676**	**0.003**
			8 h & 24 h	**0.424**	**<0.001**
		Experiment occasion	8 h	**0.507**	**0.003**
			24 h	0.172	0.095
			8 h & 24 h	**0.282**	**0.002**
		Time point	8 h & 24 h	**0.136**	**0.039**
SCFA	Donor I	Substrate	8 h	**0.331**	**0.035**
			24 h	**0.574**	**0.004**
			8 h & 24 h	0.035	0.255
		Experiment occasion	8 h	**0.780**	**0.003**
			24 h	0.098	0.196
			8 h & 24 h	0.045	0.184
		Time point	8 h & 24 h	**0.999**	**<0.001**
	Donor II	Substrate	8 h	**0.604**	**<0.001**
			24 h	**0.681**	**0.001**
			8 h & 24 h	**0.269**	**0.005**
		Experiment occasion	8 h	0.013	0.364
			24 h	-0.046	0.502
			8 h & 24 h	0.007	0.331
		Time point	8 h & 24 h	**0.331**	**0.002**

Supplementary Table S2. Mannose and galactose residue recovery of the respective combined insoluble (IS) and soluble (S) sugar residues in the fermentation substrates (mean ±SD).

	Oats, recovery-%		Rye, recovery-%		Wheat, recovery-%	
	Donor I	Donor II	Donor I	Donor II	Donor I	Donor II
IS mannose	26.51 ±6.39	17.87 ±1.83	26.40 ±5.64	12.22 ±2.80	11.91 ±2.24	6.58 ±1.44
S mannose	3.08 ±0.09	2.42 ±0.35	2.72 ±0.27	2.40 ±0.12	2.65 ±0.25	1.83 ±0.42
IS galactose	56.75 ±0.63	54.65 ±5.59	70.69 ±3.35	71.91 ±9.65	43.11 ±4.55	35.80 ±10.0
S galactose	26.64 ±2.20	17.07 ±2.18	6.55 ±1.02	11.36 ±0.54	21.0 ±4.47	13.10 ±3.29

Supplementary Table S3: Bread ingredient lists and baking information.

Bread type	Ingredients	Baking date	Bakery
Oats	Water, whole grain oats (flour, flakes and groats), sunflower seed, refined oat flour, pumpkin seed, rapeseed oil, psyllium, dried starter (oats), oat fiber, yeast, salt, preservative (E200), thickening agent (E412).<br>Oats 100% of grain ingredients.	10/05/2018	Fazer Leipomot Oy, Lahti, Finland
Rye	Whole grain rye flour, whole grain wheat flour, refined wheat flour, water, sourdough from whole grain rye flour, rye fiber, yeast, salt, barley malt extract.<br>Rye 58% of grain ingredients	10/01/2018	Fazer Bageri AB, Lidköping, Sweden
Wheat	Water, refined wheat flour, refined oat flour, malt, salt, yeast.	03/21/2019	Fazer Bageri AB, Lidköping, Sweden

Supplementary Table S4. Inclusion and exclusion criteria for study subjects.

Inclusion criteria
Age 18-65 years
Body mass index (BMI) 18,5-30 kg/m^2
Working or studying at SLU in Campus Ultuna
Available to deliver samples during the study experiment period
**Exclusion criteria**
Use of internal antimicrobial medication during the past 3 months before the study
Irregular bowel function
Type 2 diabetes, metabolic syndrome or an inflammatory or a functional disease of gastrointestinal tract
Suspicion or a diagnosis of a following infectious disease: HIV, hepatitis or Salmonella during the past 6 months
Planning to change diet considerably during the study time

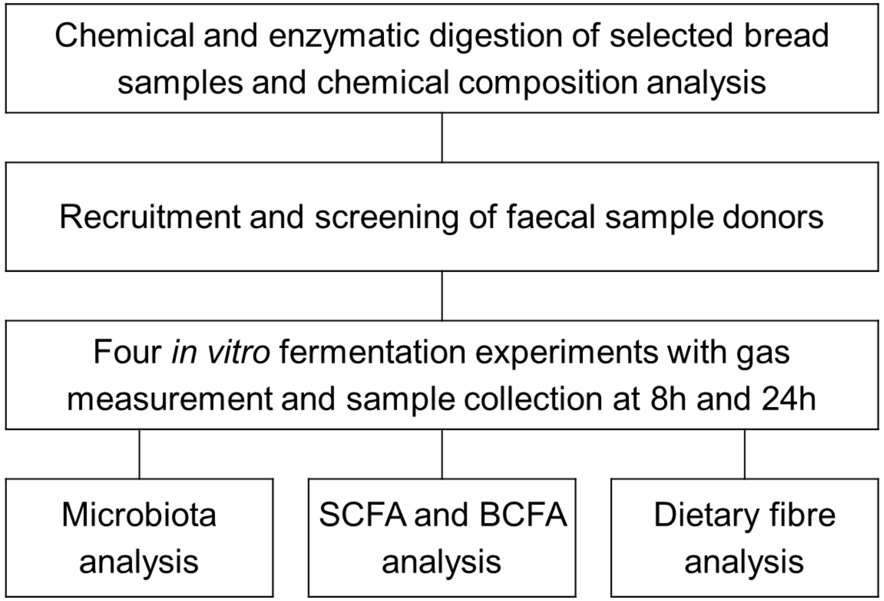

Supplementary Figure S1. Simplified study outline.

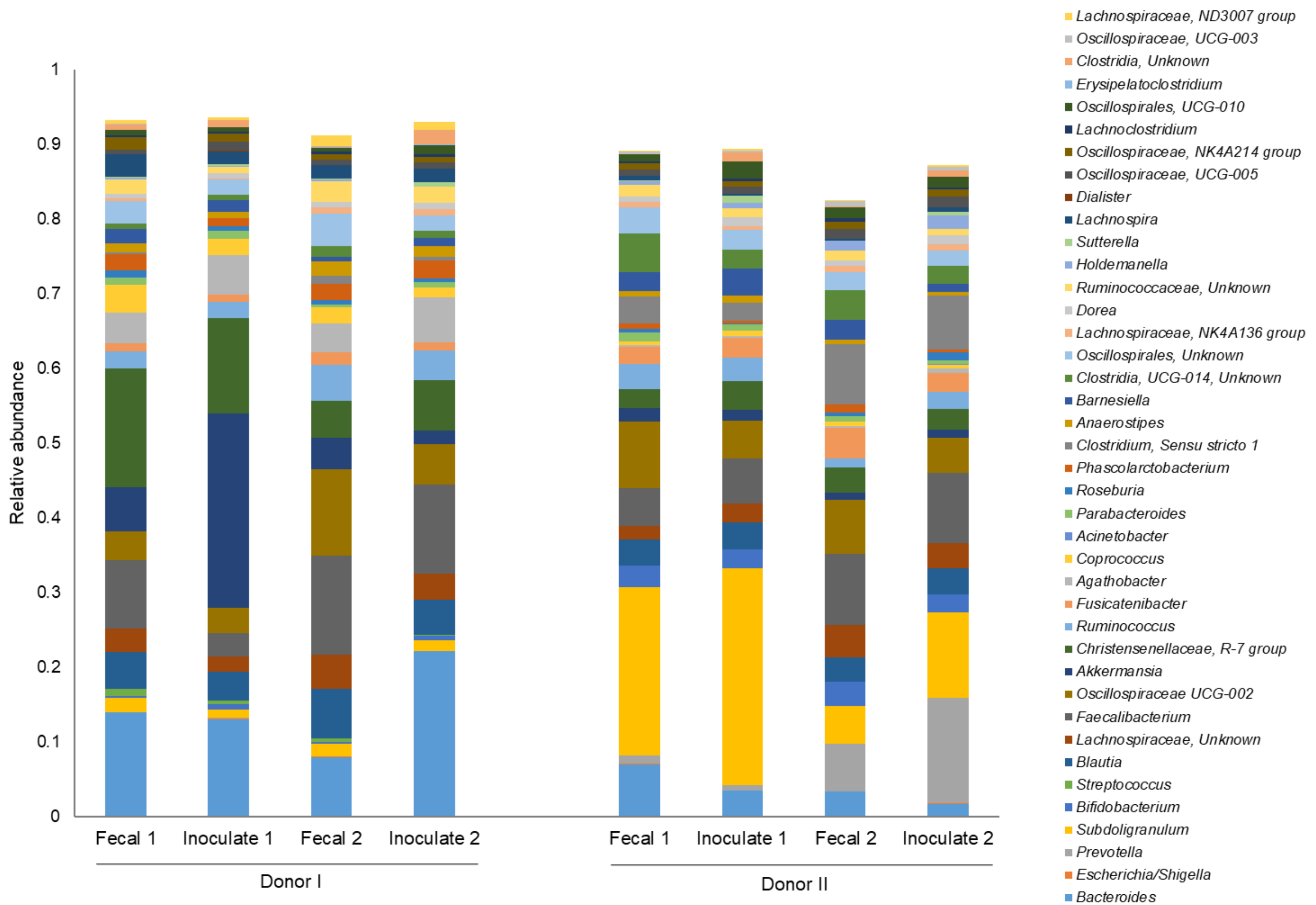

Supplementary Figure S2. The 40 most abundant microbial genera in fecal samples and inoculates used in the fermentation experiments. In each donor, the same number in fecal and inoculate indicate same experiment occasion.

Supplementary method: Modifications in the fructan content analysis method with a K-FRUC kit

1) Pre-treatment with α-galactosidase was carried out to remove galactosyl-sucrose oligosaccharides;

2) The extraction step was scaled down to 100 mg of sample and 10 mL deionized water with incubation in a glass tube at 80 °C for 20 min; and

3) The filtration step was replaced with centrifugation of 1 mL of sample for 15 min at 10 500 × g, and the supernatant was used for analysis.

Supplementary Method: 16S rRNA gene sequencing of fermentation samples and fecal samples

DNA was extracted with a NucleoSpin® 96 Soil kit (Macherey-Nagel GmbH & Co. KG, Düren, Germany) with bead beating horizontally at 2700 rpm for 5 min on a Vortex-Genie 2 (Scientific Industries Inc., Bohemia, NY, USA). A minimum of one positive control (ZymoBIOMICS™ Microbial Community Standard, Zymo Research Co., Irvine, CA, USA) and one negative control was included with each batch of samples. Polymerase chain reaction (PCR) was conducted using universal bacterial 16S rRNA gene primers targeting the V3-V4 region; the forward primer S-D-Bact-0341-b-S-17 and the reverse primer S-D-Bact-0785-a-A-21 [1], with Illumina adapters attached. The cycling conditions used in PCR were as follows: Initial denaturation at 98 °C for 30 s, followed by 25 cycles at 98° C for 10 s, at 55 °C for 20 s, and at 72 °C for 20 s, with a final elongation step at 72 °C for 5 min. Amplification was verified by gel electrophoresis. Index tags were added in a subsequent PCR using the Nextera Index Kit V2 (Illumina Inc., San Diego, CA, USA) with the PCR cycling conditions described above, but with only eight cycles instead of 25. Products from the second PCR were pooled based on band intensity and the resulting library was cleaned with AMPure XP magnetic beads (Beckman Coulter Ltd, Bread, CA, USA). The DNA concentration in pooled libraries was measured using an AccuLite 470 fluorometer (Biotium Inc., San Francisco, CA, USA). Sequencing was performed on an Illumina MiSeq desktop sequencer using the MiSeq Reagent Kit V3 (Illumina Inc., San Diego, CA, USA) for 2 × 300 bp paired-end sequencing. An adjusted DADA2 pipeline was used for bioinformatics processing of the sequence data into the amplicon sequence variant (ASV) abundance table, performed as described earlier [2]. Taxonomic assignment of the different ASVs detected was carried out using a naive Bayesian classifier algorithm comparing the ASV sequences to the SILVA reference database (version 138) [3].

Supplementary method: Modifications in the analysis method of dietary fiber content and composition in fermentation samples

1) Sample amount for insoluble fiber samples was 20 mg and for soluble samples 100 mg;

2) Analysis started directly from the hydrolysis step, scaled down to 1/12 for insoluble fiber samples and to a volume of 3 mL for the soluble fiber samples;

3) The amount of myoinositol was 0.5 mg; and

4) Sample volume was not standardized after the hydrolysis step, and thus samples were weighed and mass was then converted to volume.

References

1 Klindworth A., *et al*. Evaluation of general 16S ribosomal RNA gene PCR primers for classical and next-generation sequencing-based diversity studies. *Nucleic Acids Res*. **41,** e1 doi:10.1093/nar/gks808 (2013).

2 Callahan B.J., *et al*. DADA2: High-resolution sample inference from Illumina amplicon data. *Nat Methods.* **13**, 581-583, doi:10.1038/nmeth.3869 (2016)

3 Quast C., et al.. The SILVA ribosomal RNA gene database project: improved data processing and web-based tools. *Nucleic Acids Res.* 41, D590-596, doi: 10.1093/nar/gks1219 (2013)
